# How the Immune System Learns

**DOI:** 10.1103/znt6-6n38

**Published:** 2026-05-13

**Authors:** Arup K. Chakraborty, Anupama Jayaraman, Daniel P. Newton

**Affiliations:** 1 Department of Chemical Engineering, Massachusetts Institute of Technology, Cambridge, Massachusetts 02139, USA; 2 Department of Physics, Massachusetts Institute of Technology, Cambridge, Massachusetts 02139, USA; 3 Department of Chemistry, Massachusetts Institute of Technology, Cambridge, Massachusetts 02139, USA; 4 Institute for Medical Engineering & Science, Massachusetts Institute of Technology, Cambridge, Massachusetts 02139, USA; 5 Ragon Institute of Massachusetts General Brigham, Massachusetts Institute of Technology & Harvard University, Cambridge, Massachusetts 02139, USA; 6 Department of Physics, Harvard University, Cambridge, Massachusetts 02138, USA

## Abstract

The adaptive immune system can mount pathogen-specific responses against a vast world of evolving pathogens (antigens). The humoral immune system is a part of adaptive immunity. It has the remarkable ability to learn about an antigen after exposure to it through a mutation-selection evolutionary process, resulting in more potent responses than those that emerge initially. Recent studies have revealed that upon reexposure to antigens, feedback regulation mediated by past responses steers the evolutionary learning algorithm to generate new responses. This feature enables the immune system to learn to generalize responses to target unseen variants of the original pathogen upon “training” with a limited number of samples. We summarize these new findings and suggest new questions that emerge from them. This is the right time to address these questions by bringing together statistical physics, machine learning, immunology, and evolutionary biology.

## I. INTRODUCTION

The immune system of vertebrates is composed of two interlinked parts, the innate and adaptive immune systems. The cells and molecules of the innate immune system are the first responders to infection. The innate immune system controls pathogen growth, classifies the type of infection (viral, bacterial, etc.), and instructs the adaptive immune system to mount appropriate protective responses. However, innate immune responses are not specific to any particular pathogen [1]. The adaptive immune system is able to mount pathogen-specific responses against diverse evolving pathogens. It also establishes a memory of past pathogen exposures, enabling a rapid and robust response upon reexposure to the same pathogen. This pathogen-specific memory is the basis for vaccination, which aims to elicit protective memory responses. The adaptive immune system has two principal arms, one composed of T cells and the other B cells. The humoral immune system, composed of B cells and their products, has the remarkable ability to learn about new pathogens or vaccine components (antigens) and generate responses that are more effective than those that initially emerge [2]. This perspective is focused on recent advances in our understanding of how the humoral immune system learns. Fundamental studies of these mechanisms represent an exciting intersection of statistical physics, evolutionary biology, learning algorithms, and immunology. The knowledge thus generated could potentially help cure and prevent disease.

## II. HOW THE HUMORAL IMMUNE SYSTEM LEARNS

**FIG. 1.**
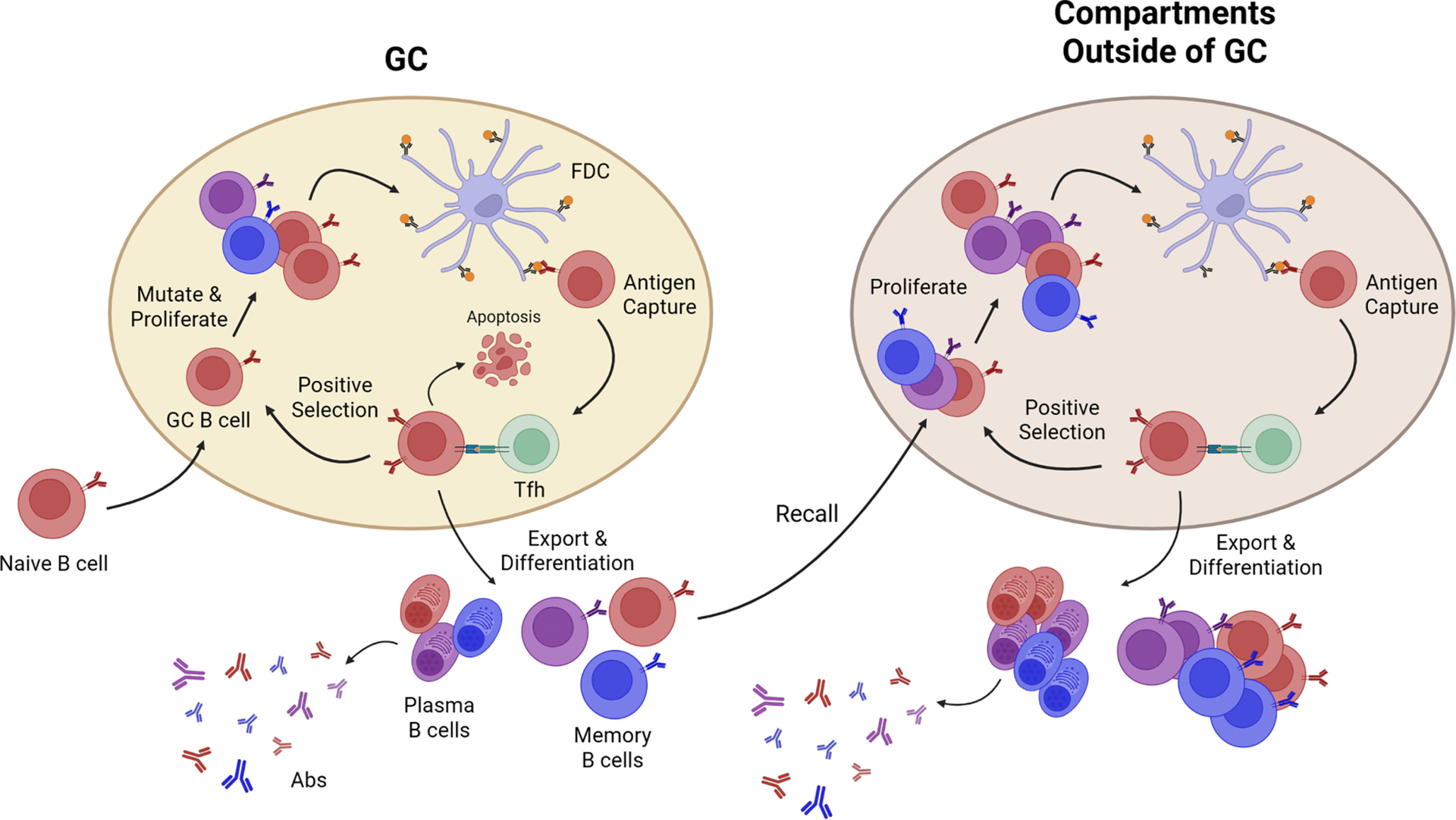
B cell dynamics in GCs and compartments outside GCs. After the first antigen exposure (left), naïve B cells that bind moderately strongly to the antigen and receive Tfh help can enter germinal centers (GCs). Therein, GC B cells proliferate and undergo repeated rounds of BCR mutation and selection. In each cycle some positively selected B cells exit the GC as memory or Ab-secreting plasma cells (see text). Upon subsequent antigen exposures, memory cells enter compartments outside of GCs (right) and undergo rapid affinity-dependent expansion with negligible mutation, and the majority exit and differentiate into Ab-secreting cells that produce a wave of antibodies. In these compartments, antigen is presented on various antigen-presenting cells, such as macrophages and FDCs. New GCs also form upon antigen reexposure to produce new responses over much longer time scales.

Humans have tens to hundreds of billions of B cells, each of which expresses a B cell receptor (BCR) on its surface. A huge diversity (~10^8^–10^11^) of BCR sequences is generated during B cell formation and development, so the BCR on any B cell is likely distinct from those on most other B cells [3]. Upon antigen exposure, naïve B cells with BCRs that bind moderately strongly (dissociation constant *K*_*d*_ ≲ 10^−6^ M in mice [4]) to the antigen can get activated and enter structures in lymph nodes called germinal centers (GCs) [2]. In GCs, B cells learn about the antigen through a Darwinian evolutionary process (Fig. 1, left panel).

Activated B cells proliferate in GCs, and an enzyme expressed in GC B cells introduces mutations at a high rate (~10^−3^ per base pair per replication) in the antigen-binding region of the BCR [5]. Most affinity-changing mutations are deleterious; in mice, ~5% of affinity-changing mutations are beneficial [6]. These B cells, some of which have mutated BCRs, then interact with antigen presented on follicular dendritic cells (FDC) in GCs [2]. When the B cell’s cytoskeleton retracts, a pulling force is exerted on the BCR. If the BCR binds sufficiently strongly to the antigen, then this force extracts the antigen from the FDC and the B cell internalizes it [7]. Internalized antigen is then chopped into short peptides that can bind to proteins encoded by the major histocompatibility (MHC) genes (known as human leukocyte antigen in humans). The resulting peptide (p)-MHC complexes are displayed on the B cell surface and they can interact with receptors on GC-resident T follicular helper (Tfh) cells. A productive interaction between Tfh and pMHC molecules on a B cell results in positive selection. Without this survival signal from Tfh, B cells die. B cells with BCRs that bind more strongly to the antigen are likely to internalize more antigen, display more pMHC molecules on their surface, and outcompete other B cells for productive Tfh interactions. Enhanced Tfh levels promote survival of B cells that bind to antigen more weakly [8]. A minor fraction of the positively selected cells exits GCs as memory B cells or plasma cells [2,9]. Plasma cells secrete soluble forms of the BCR called antibodies (Abs). Abs can then bind to antigen and prevent infection of new cells and help clear the pathogen in various ways [10]. Most positively selected B cells are recycled for further rounds of mutation and selection, thus increasing the response’s potency over time [2].

Upon reexposure to the same antigen, existing memory B cells rapidly expand in an affinity-dependent fashion in compartments outside GCs by processes like those that occur in GCs [11]. However, there is little to no mutation, and the majority of exiting B cells are plasma cells that secrete Abs (Fig. 1, right panel) [12]. These Abs generated by boosting past responses are generated fast enough to provide protection; for example, the Ab response peaks in about 6 days upon receiving a second dose of COVID vaccines [13]. Upon antigen reexposure, new (secondary) GCs form, allowing B cells to learn about the antigen over longer timescales (weeks to months in humans).

The processes described above and depicted in Fig. 1 are stochastic dynamical processes driven far from equilibrium. Various physics-based computational and theoretical approaches have been developed to study these processes in synergy with experimental and clinical studies [8,14–20].

## III. RECENT ADVANCES IN UNDERSTANDING THE RECALL RESPONSE

Recent experimental, clinical, and computational and theoretical studies have shed new light on the recall response upon reexposure to antigen. We briefly outline a few examples and describe the common principle underlying these results.

**FIG. 2.**
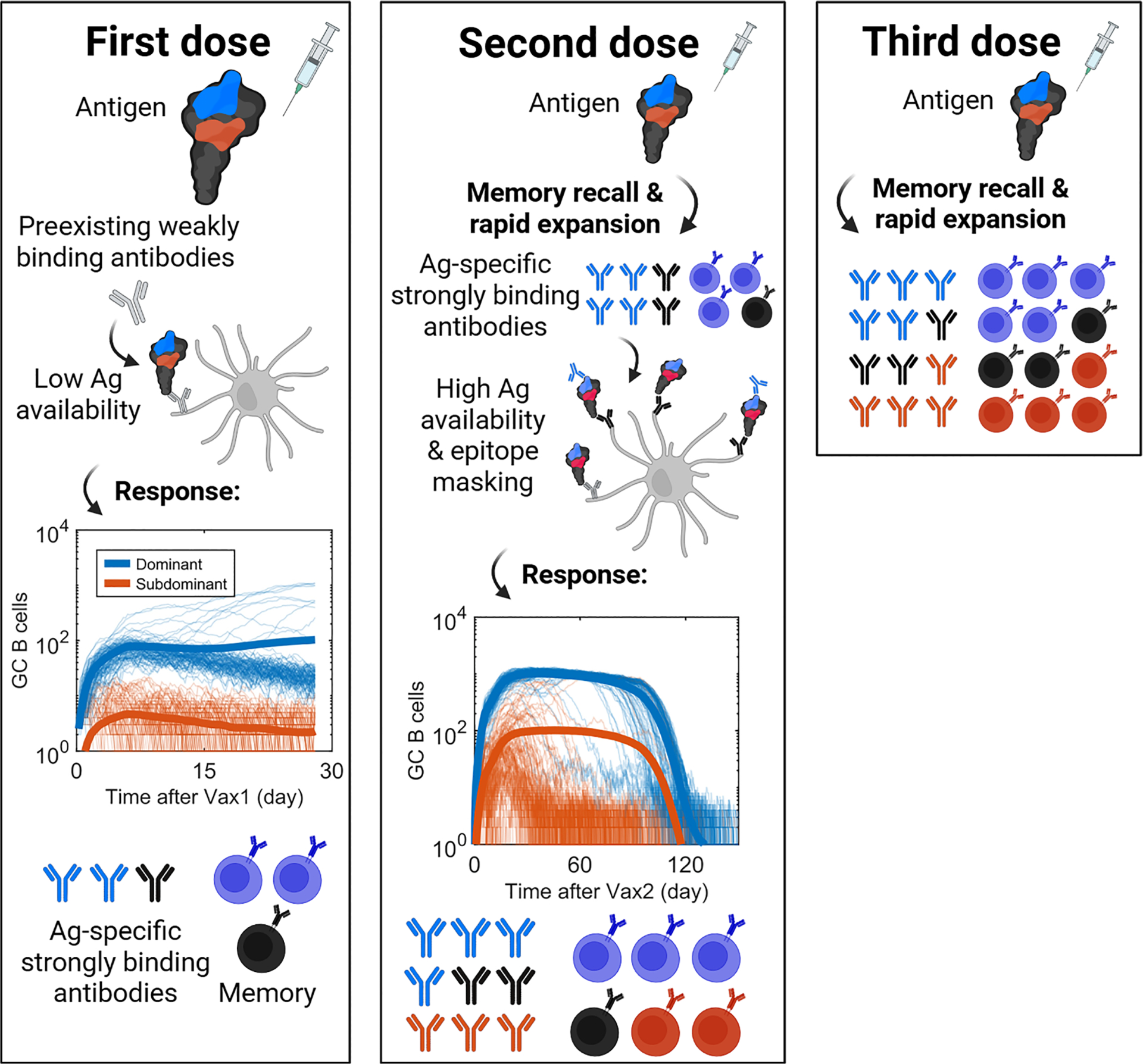
Antibody feedback upon repeated antigen exposure influences responses. After the first antigen exposure (left panel), preexisting generic weakly binding Abs deposit small amounts of antigen (Ag) to FDCs, which, as noted in text, predominantly generates an immunodominant response (blue Abs and memory B cells), but also generates responses to non-RBD epitopes (black Abs and memory B cells). After the second antigen exposure (middle panel), memory B cells produced during the first antigen exposure are rapidly expanded, leading to the production of potent Ag-specific Abs. These Abs bind to and deposit more Ag on FDCs (compared to the first exposure). Some Abs mask immunodominant (blue) epitopes (see text), resulting in a response that includes both immunodominant and subdominant responses (blue and red Abs and memory B cells). After the third Ag exposure (right), the prior response is rapidly expanded, leading to Abs that bind to immunodominant (blue) and subdominant (red), epitopes. The panels showing GC B cells as a function of time are results of computer simulations; data taken from Ref. [16].

During the COVID19 pandemic, people vaccinated with three doses of the identical vaccine encoding the spike proteins of the original Wuhan strain were better protected by Abs against Omicron, the highly mutated variant of SARS-CoV-2, than those who had received only two doses [21]. If repeated antigen exposure just boosts past responses, then how could this be so? Upon exposure to antigen, circulating Abs that bind to it deposit antigen on FDCs in GCs. During the first exposure, the available circulating Abs are generic preexisting ones that bind weakly to the antigen. So, before the antigen rapidly decays (a few days in monkeys [22]), these Abs can bind to and deposit very little antigen. This result is supported by experiments in monkeys with a different vaccine [22]. As antigen presented on FDCs drives selection processes, small amounts of antigen on FDCs represent a stringent selection limit. Thus, only the naïve B cells that bind relatively strongly to antigen compared to others can enter GCs and compete effectively. The resulting memory B cells and Abs comprise the immunodominant response (Fig. 2, left). The receptor binding domain (RBD) is a part of the SARS-CoV-2 spike that binds to receptors on human cells to infect them. Consistent with the mechanism noted above, the immunodominant Abs that bind to regions (epitopes) on the RBD exhibit only a few mutations relative to their naïve precursors [23]. After the second dose of the COVID vaccine, the Ab response generated in the first few days is the result of expansion of memory B cells produced by GCs after the first dose (Fig. 2, middle). The epitopes of immunodominant Abs that target the RBD are highly mutated in the Omicron variant [24], so the generated Abs are not effective in binding to the RBD to prevent infection by this variant [16].

**FIG. 3.**
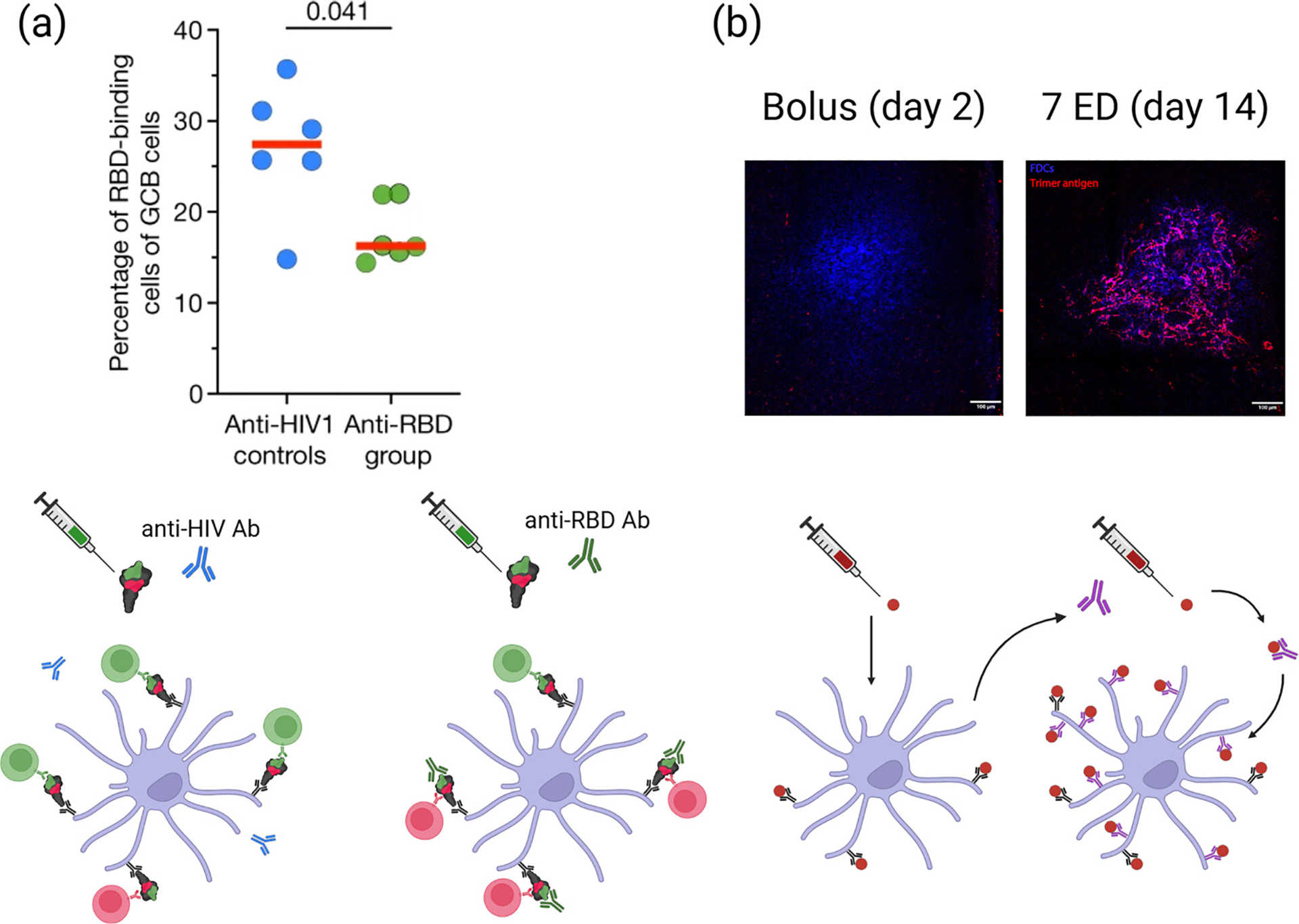
Antibody feedback demonstrated in animal models. (a) In mice, pretreatment with SARS-CoV-2 RBD-specific Abs decreases the fraction of RBD-specific GC B cells, without impacting the magnitude of the overall GC reaction. Experiments with HIV-specific Abs act as a control that should not be able to interact with the SARS-CoV-2 Ag. This suggests that the specificity of GC B cells shift to targeting alternative epitopes (pink) due to epitope masking of the epitopes targeted by exogenously added Abs (green). Data shown taken from Schaefer-Babajew *et al*. [26]. (b) In mice, lymph node imaging demonstrates that vaccination with a single dose (left) results in little antigen (Ag; red) deposited on FDCs due to the weakly binding preexisting Abs. In a 7-day extended dosing scheme (right), Abs produced from the initial doses facilitate more Ag deposition on FDCs during later doses. This result is supported by experiments in complement-deficient mice, which are unable to efficiently deposit Ag with Abs, that show small amounts of Ag on FDCs after the extended dosing scheme. Data shown taken from Bhagchandani *et al*. [27].

Upon antigen reexposure, potent antigen-specific Abs are generated that deposit much more antigen on FDC surfaces in secondary GCs, consistent with experiments in monkeys [22]. With more antigen, selection is less stringent and naïve B cells that bind more weakly to the antigen than those that developed into the immunodominant response can also enter and compete within GCs. These subdominant responses are thus promoted in GCs that form after the second shot. This is an example of feedback regulation of GCs that form upon reexposure to antigen by responses that emerged earlier. Another feedback loop, which was first noted in 1909 [25] but has only recently been highlighted and properly understood, also promotes subdominant responses. Abs specific for the immunodominant RBD epitopes can enter GCs and bind to these epitopes. This lowers the availability of these epitopes relative to the subdominant epitopes, further promoting subdominant responses in secondary GCs (Fig. 2, middle) [16]. This effect, called epitope masking, has been observed in carefully controlled experiments in mice [Fig. 3(a)] [26,28–30].

The subdominant responses can evolve over long time scales (~3 months) in GCs that form after the second shot of a COVID vaccine and the corresponding memory B cells generated become potent over time. The RBD epitopes that correspond to these subdominant responses are not highly mutated in Omicron. After the third shot, memory B cells corresponding to dominant and subdominant responses are expanded to generate a wave of corresponding Abs (Fig. 2, right). Those targeting the subdominant RBD epitopes confer protection against Omicron [16,24].

Clinical data show that repeated immunization with the 2009 pandemic strain of influenza led subjects to develop responses to historical strains after the later immunizations. Computational studies suggest that these results also reflect feedback regulation of GCs that form upon reexposures to antigen, especially epitope masking [31].

The effects of feedback on GC responses upon antigen reexposure due to epitope masking and higher antigen levels in FDCs may be leveraged in other settings. In recent years, a new vaccination protocol has been studied where instead of administering the antigen as a single injection, the same total amount of antigen is administered in several doses over 2 weeks, with the quantity of antigen increasing in each dose. This dosing pattern mimics infection with a replicating pathogen and results in a stronger and broader Ab response in mice and monkeys [32–34], and clinical trials are underway. Imaging studies in mice demonstrate that extended dosing patterns result in more antigen on FDCs [Fig. 3(b)] and that this effect is dependent on Abs generated in response to the initial antigen doses. Based on a mechanistic understanding of this effect and epitope masking, computational studies designed a more pragmatic two-dose extended dosing pattern predicted to result in humoral responses comparable to extended dosing with many (seven) doses, which has been positively tested in mice [27].

Computational studies suggest that epitope masking underlies observations in clinical studies pertinent to efforts to develop strategies to cure people living with HIV [35–38]. These studies propose that continuous exposure to diverse strains of HIV in GCs, when viral load is suppressed by therapeutic agents, allows for the formation of a polyclonal net of Abs that target diverse epitopes on the HIV spike proteins. After the most immunodominant response emerges, the corresponding Abs could mask the dominant epitope and allow the next response in the immunodominance hierarchy to emerge. This process could go on to generate a polyclonal net of Abs targeting many different epitopes, which would be difficult to evade by viral mutations. Emerging data seem to support these ideas in some people who can maintain control of viral load for long times post-therapy without interventions [35,39–41].

Taken together, the recent studies outlined above illustrate the following concept. Feedback regulation enables initially low-probability GC evolutionary trajectories, such as those that target subdominant epitopes, to become more likely upon repeated antigen exposure. Thus, feedback regulation enables the learning algorithm represented by the humoral immune system to respond to unseen variants upon “training” with a limited number of samples. This is an example of the general phenomenon of interplay between evolution and ecology: the evolution of B cells in GCs that form upon initial antigen exposure changes the environment (ecology) by generating Abs, which in turn influence evolution in secondary GCs. Ecological-evolutionary feedback is also well studied in predator-prey dynamics [42], microbes [43,44], and tumors [45].

## IV. NEW QUESTIONS

The recent findings described above lead to interesting new questions at the intersection of statistical physics, evolutionary biology, and learning theory. While epitope masking might seem inevitable, it is conceivable that the system might have evolved mechanisms to inhibit antibody entry into GCs if selection forces favored this. So, one question that emerges is the following: What might have been the selection force over millennia of evolution that led to epitope masking becoming a part of the learning algorithm represented by humoral immunity? A hypothesis is that repeated exposure to the same virus likely led to subsequent infections with variant strains. The ability to generalize responses to protect against the variants upon training with a limited number of early exposures to the original virus would maximize long-term rewards. In response to this selection pressure, epitope masking may have evolved as a way to use past Ab responses to enable generalizing responses. Is this hypothesis plausible?

This hypothesis immediately raises another question. Evolution of a mechanism to generalize responses upon repeated exposure to the same antigen was constrained to use existing Abs for epitope masking, but we are not similarly constrained. For example, along with vaccine antigens, we could passively administer Abs that target specific epitopes on the antigen. Such interventions could allow evolutionary trajectories in GCs to be steered for desirable outcomes beyond what can be achieved by natural epitope masking alone. What is the optimal strategy for administering such exogenous interventions along with a few shots of the same or variant antigens that would allow the development of a polyclonal net of Abs targeting different epitopes on an antigen? Such an Ab net might be effective at protecting against several strains of a mutable pathogen. For example, such a vaccination strategy may be able to confer protection for many seasons of influenza. More generally, thinking about such questions could teach us fundamentally new aspects of the humoral immune response and how GC evolutionary strategies can be steered by the interplay between ecology and evolution.

Escalating temporal patterns of antigen dosing results in long-running GCs [32,33] where the effects of epitope masking can be manifested to impact the humoral response. What happens when a variant antigen (either by itself or along with an exogenous intervention) is administered while there is still an ongoing GC due to extended dosing? More generally, the issues identified in the preceding paragraph and this one suggest the following question: How does the combination of antigen identity, order, and timing of administration interact to influence the learning process through feedback regulation? Addressing these questions may also clarify the role of other immune factors, such as Tfh cells, in determining evolutionary trajectories in GCs.

Recent findings also motivate broader fundamental questions. Is there a theoretical limit to how much the immune system can learn about a mutable pathogen through feedback regulation? When subject to a dominant immune response, mutable pathogens can evolve to evade the response, leading to the emergence of new immune responses. How does such coevolution between a pathogen and the humoral response and feedback regulation affect the learning process and the theoretical limit of learnability (should such a limit exist)?

## V. THE PATH FORWARD

During their evolution in GCs, B cells balance the trade-off between mutation in the BCR sequence space (exploration) and affinity-based competition (exploitation). This type of learning algorithm is well studied in artificial intelligence contexts such as reinforcement learning [46]. Therefore, a promising way to address the identified questions may be by drawing analogies between how machines learn and how the immune system learns. With this framing, Ab feedback mechanisms essentially tune the balance between exploration and exploitation. While it is difficult to obtain large volumes of data on GC processes, physics-based models that capture the essential features of the humoral immune response could be good proxies that can generate such data for the purposes of obtaining insights using machine learning and artificial intelligence tools.

Some exciting concepts may also be uncovered about how different learning algorithms interact to determine outcomes. For example, pathogen populations evolve in response to human immune pressure [47]. Extending analogies between how machines learn and the immune system learns to study immune-pathogen coevolution [48,49] could clarify how three learning algorithms—immune learning, pathogen learning and machine learning—interact with each other.

If there is a theoretical limit to how much the immune system can learn about a mutable pathogen through feedback regulation, then such a limit would presumably depend on ecological feedback and evolutionary pressures, potentially motivating a direct mapping to classical eco-evo theory [50].

In summary, recent studies show that the immune system learns in interesting ways through feedback regulation upon reexposures to antigen. Many exciting questions now suggest themselves and this is the right time to address them by combining physics-based computations (stochastic B cell evolutionary trajectories) with machine learning algorithms (e.g., reinforcement learning to find adaptive control strategies) and experiments in animal models. The resulting knowledge could help guide new ways to cure and prevent disease.

## Data Availability

There are no publicly available research data or software supporting this manuscript. Requests for further information or data should be sent to the authors.
